# Optimal Designs of Staggered Dean Vortex Micromixers

**DOI:** 10.3390/ijms12063500

**Published:** 2011-06-03

**Authors:** Jyh Jian Chen, Chun Huei Chen, Shian Ruei Shie

**Affiliations:** Department of Biomechatronics Engineering, National Pingtung University of Science and Technology, 1, Shuefu Road, Neipu, Pingtung 91201, Taiwan; E-Mails: chaucer1114@gmail.com (C.H.C.); roni@ms13.url.com.tw (S.R.S.)

**Keywords:** lamination micromixer, confocal microscope, pH indicator, secondary flow, staggered microchannel, Dean vortex

## Abstract

A novel parallel laminar micromixer with a two-dimensional staggered Dean Vortex micromixer is optimized and fabricated in our study. Dean vortices induced by centrifugal forces in curved rectangular channels cause fluids to produce secondary flows. The split-and-recombination (SAR) structures of the flow channels and the impinging effects result in the reduction of the diffusion distance of two fluids. Three different designs of a curved channel micromixer are introduced to evaluate the mixing performance of the designed micromixer. Mixing performances are demonstrated by means of a pH indicator using an optical microscope and fluorescent particles via a confocal microscope at different flow rates corresponding to Reynolds numbers (*Re*) ranging from 0.5 to 50. The comparison between the experimental data and numerical results shows a very reasonable agreement. At a *Re* of 50, the mixing length at the sixth segment, corresponding to the downstream distance of 21.0 mm, can be achieved in a distance 4 times shorter than when the *Re* equals 1. An optimization of this micromixer is performed with two geometric parameters. These are the angle between the lines from the center to two intersections of two consecutive curved channels, *θ*, and the angle between two lines of the centers of three consecutive curved channels, *ϕ*. It can be found that the maximal mixing index is related to the maximal value of the sum of *θ* and *ϕ*, which is equal to 139.82°.

## 1. Introduction

Among the rapidly-developing microfludic devices, such as micro heat exchangers, microreactors, and DNA analyzers, a micromixer plays an important role in bio-analytical and chemical applications. Recently, an increasing number of researchers have integrated micromixers into their analysis systems. The synthesis of active ingredients for the pharmaceutical industry [1] and the studies of immuno-magnetic cell sorting [2] are two examples. Many researches on the miniaturization of chemical analysis systems have considered the reactions of solutions in a microsystem. The integration of electrochemical detection approaches into microfluidics has a simple hardware requirement. A micromixer was incorporated into a hybridization chamber to aid in mixing during loading [3]. The two-stage copolymerizations are carried out without any intermediate purification step. The main advantage using microstructured mixers is their ability to achieve a rapid and efficient mixing between two fluids [4]. The wide applications of micromixers have garnered a lot of attention, and the micromixer has been moved from the sub-systems of micro-TAS to one of the crucial components of MEMS. Fast mixing can decrease analysis time and improve reaction efficiency in industrial applications. Mixing of the liquid flows in microchannels is difficult because the flows belong to the regime of laminar flow and mixing in microfluidic devices is dominated by molecular diffusion. However, if mixing is poor, the reaction may be incomplete, thus degrading the functions of systems.

Over the last decade, many studies have investigated passive micromixers. The manufacture of chaotic micromixers and serial lamination micromixers increases the complexity of the micro-fabrication process. Parallel lamination mixers with simple two-dimensional structures are fabricated without difficulty, and mixing in such laminar flows can be easily enhanced. A micromixer based on the principle of distributive mixing was presented by Hong *et al.* [5]. An in-plane micromixer using Tesla valve structures for the Coanda effect was designed and a Poiseuille flow was created in the normal direction of the flow to achieve thorough mixing. A passive micromixer using a recycle flow was devised by Jeon *et al.* [6]. A recycle flow was introduced from side channels to the inlet flow and used to improve the mixing performance of two fluids.

Transverse Dean Flows develop in curved channels due to the action of centrifugal forces. This provides enhanced heat transfer in various applications [7–9]. In these channels, curvature amplifies the lateral transportation that drives a secondary cross-section flow. Mixing can also be improved when liquids flow in such curved microchannels. The mixing improved by Dean vortex flows of various configurations of microchannels was demonstrated and the radius of curvature was emphasized. Howell *et al.* [10] fabricated a three-quarter ring-shaped channel. The dye profiles across the channel at several positions were depicted to express the strength of secondary flows. Results also showed that the bulk of the flow shifts to the outside and then appears to split into two separate streams. This implied that the large aspect ratio channel was preferable for a mixer. Yamaguchi *et al.* [11] observed the flow patterns in a microchannel with constant hair-pin curves. The results revealed that the interface configuration was affected by the curvature radius. The increased interface area of liquids promoted a mass transfer. Jiang *et al.* [12] presented a channel comprised of four three-quarter ring-shaped channels. At Dean numbers around 100, the interface lengths obtained from the particle tracking method increased exponentially in a downstream direction. Kockmann *et al.* [13] presented the concentration distribution in a channel with a 90° bend for Reynolds numbers (Re) ranging from 120 to 300 by CFD software. The mass transportation of liquid fluids was increased with increasing values of *Re*.

On a relatively smaller scale, flow in curved channels can be found in heat exchangers, chemical separation systems, and membrane filtration systems, *etc.* To improve the mass transportation, however, the flow rate inside the curved channel should be very large and the corresponding *Re* should be larger than 120. The fairly large *Re* is beyond the range of conditions realistically achievable in many microfluidic devices, so the flows with smaller *Re* in curved channels were split, recombined and studied in order to enhance mixing. Sudarsan and Ugaz [14] demonstrated a planar split-and-recombination (SAR) micromixer experimentally. Parallel liquid streams first travelled through a curved segment that induced simultaneous 90° rotations in the upper and lower halves of the channel, at which point the flow was spilt into multiple streams that continued along curved trajectories such that each individual split stream experienced a second pair of 90° rotations. Mixing and flow characterization studies were performed by using cross-sectional confocal imaging. Mouza *et al.* [15] experimentally illustrated a micromixer comprised of a semicircular curved channel and a SAR unit consisting of two semicircular microchannels that form a circle. The measured mixing time as a function of Dean number was shown. At Dean numbers less than 150, the addition of SAR features considerably promoted fluid mixing.

The mixing characteristics of the liquids traveling through curved channels were investigated in previous works. In all of the aforementioned studies, the effects of staggered geometries on the flow characteristics inside curved channels were not investigated. However, the uneven split of two fluids inside staggered channels does improve the mixing performance. Dean Vortex micromixers have been proposed to increase mixing efficiency; its centrifugal force is responsible for producing the secondary flows in curved channels. For the mixing experiment, two different types of experiments, by means of phenolphthalein and via a confocal microscope, have never been carried out in the same study. Characterization of the mixing performance of mixing devices using CFD simulations has been described in many publications. But apparently there have been no previous computational assessments among the SAR micromixers with curved channels. In addition to the researchers of novel configuration design, it is necessary to suggest the optimal design guidelines on micromixers [16]. To date, systematic techniques of numerical optimization have not yet been applied to Dean Vortex micromixers. Therefore, optimizing the geometric parameters of curved microchannels would effectively increase mixing.

In previous studies, some researchers considered in-plane micromixers using SAR structures for the Coanda effect [5,6]. Fluids tend to flow to the angled channel due to the Coanda effect. A strong impact around the sub-channel is produced. On the other hand, Dean Vortex micromixers with curved channels were considered in some works [10–13]. Centrifugal forces amplify the lateral transportations in curved channels that drive secondary flows. The increased interface area of liquids promotes a mass transfer. In the present work, the combination of curved channels and SAR structures is presented. A comprehensive analysis in a parallel lamination micromixer with two-dimensional curved rectangular channels is performed to examine the mass transportations and flow fields inside a staggered curved microchannel. Geometric optimization is carried out with mixing indexes by considering two geometric design variables. The computational fluid dynamics package is utilized to solve the three-dimensional flow fields and the mixing of two fluids. Two different kinds of mixing experiments by means of a pH indicator and fluorescent particles are performed. The cross-sectional concentration distributions, the particle trajectories and the mixing indices are utilized to examine the mixing and flow characteristics inside staggered Dean Vortex micromixers at *Re* ranging from 0.5 to 50. Furthermore, we compare the experimental mixing performance to the simulated results.

## 2. Mathematical Model and Numerical Methodology

**Figure 1:**
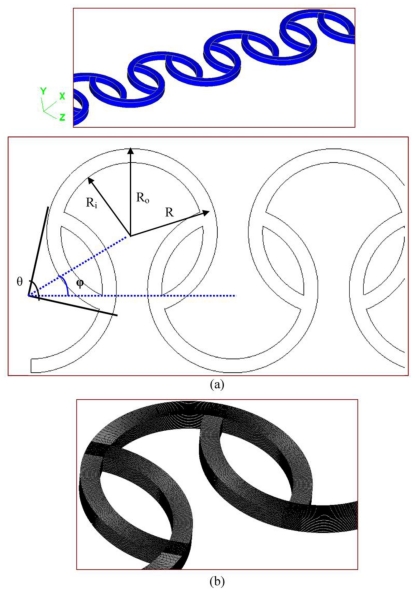
(**a**) The schematic diagram and (**b**) the grid system of the simulation domain.

A schematic diagram of the staggered Dean Vortex micromixer is shown in Figure 1. Each segment of the micromixer is comprised of 3 staggered curved channels (SCC) which are three-eighth ring-shaped and three-quarter ring-shaped with uniform width. We consider two design variables; these are the angle between the lines from the center to two intersections of two consecutive curved channels, *θ*, and the angle between two lines of the centers of three consecutive curved channels, *ϕ*. The mixing characteristics of two liquids entering the staggered microchannels, subjected to a specific inlet pumping velocity at various values of geometrical and/or operational parameters, are examined. The flow of an incompressible Newtonian liquid in microchannels can generally be depicted by the following three equations. The equations to be solved are the continuity equation, the Navier-Stokes equations, and the convection-diffusion equation, respectively. The non-slip and non-penetration conditions are usually encountered at solid surfaces.

(1) $$\nabla \cdot \vec{U}=0$$

(2) $$\rho \vec{U}\cdot \nabla \vec{U}=-\nabla P+\mu \nabla^{2}\vec{U}$$

(3) $$\rho \vec{U}\cdot \nabla \varphi =D\nabla^{2}\varphi$$

where *U⃗* is the fluid velocity vector, ρ is the density, P is the pressure, *μ* is the dynamic viscosity, *φ* is the mass concentration and D is the mass diffusivity. Equation (3) must be solved together with Equations (1) and (2) in order to achieve computational coupling between the velocity field solution and the concentration distribution.

The dimensionless groups characterizing the flows are the Reynolds number, which expresses the relative magnitudes of inertial force to viscous force:

(4) $$Re={UD}_{H}/v$$

where *U*, *D_H_*, and *v* denote the velocity, the hydraulic diameter, and the kinematic viscosity, respectively. The Dean number expresses the relative magnitudes of inertial centrifugal force to viscous force:

(5) $$K=Re{\left(D_{H}/R\right)}^{0.5}$$

where *R* is the radius of curvature of channel. And the Péclet number, which is the relative importance of mass transfer by convection compared with mass transfer by diffusion:

(6) $$Pe={UD}_{H}/D$$

In the simulations and experiments, the *Re* range from 0.5 to 50 corresponding to *K* from 0.22 to 22.36 and *Pe* from 1.25 × 10^3^ to 1.25 × 10^5^.

A commercial CFD package (CFD-ACE+^TM^, ESI, Huntsville, AL, USA) which uses a finite volume approach is utilized to analyze the three-dimensional flow and mixing in the micromixers. A nonlinear steady-state algorithm is used for hydrodynamic calculations, and a linear steady-state algorithm is used to solve the diffusion-convection equation. A three-dimensional structured grid system is created for the full model, and the SIMPLEC method is used for pressure-velocity coupling. The spatial discretizations for the convection terms are performed using a second-order upwind scheme with a limiter and the spatial discretizations for the diffusion-like terms are then presented applying a second-order central difference scheme. A fixed-velocity condition is set at the mixer inlet; the boundary condition at the outlet is a fixed pressure. The concentration of the species is normalized to 1 for the inlet on the right side, and 0 for the inlet on the left side. The fluid properties are set at the physical and thermodynamic properties of water at 25 °C and are used in our simulations unless noted otherwise. The algebraic multigrid (AMG) solver is utilized for pressure corrections, and the conjugates gradient squared (CGS) and preconditioning (Pre) solvers are utilized for velocity and species corrections. The inertial relaxation for velocities is set to 0.1, and the linear relaxation for pressure is set to 0.5. The solution is considered converged when the relative errors of all independent variables are less than 10^−4^ between successive sweeps.

In the design of any micromixer, one of the most important performance parameters is a measure of mixing intensity. The mixing intensity is quantified by calculating the mass concentration distribution. The uniformity of mixing at sampled sections is assessed by determining the mixing index MI, which is defined as

(7) $$MI=1-\frac{\int_{A}|I-I_{ave}|\text{d}A}{\int_{A_{0}}|I-I_{ave}|\text{d}A}$$

where *I* is the local concentration value (between 0 and 1) on the selected section *A*, *I*_0_ is the local concentration value at the inlet plane *A*_0_ and *I_ave_* is the averaged value of the local concentration over the selected section. The mixing index MI range is from 0 for no mixing to 1 for complete mixing.

**Figure 2:**
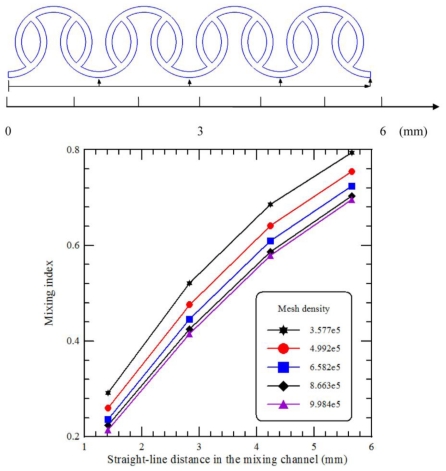
Grid-independency test.

The numerical simulation is not free from numerical diffusion errors. In Eulerian simulations, the continuous differential equations of motion are deconstructed into discrete algebraic equations. These discrete equations are generally more diffusive than the original differential equations. If liquid fluids flow diagonally through the simulated grid, then the numerical effect takes the form of an extra high diffusion rate. In the proposed grid systems, meshing is generally aligned in the flow direction in the computational domain, shown in Figure 1b. In order to obtain mesh-independent results from the simulations, a preliminary mesh size sensitivity analysis is carried out. Mesh density is increased continuously until it has minimal effects on the mixing index at the specific cross sections. Figure 2 shows the values of the mixing index at different cross-sectional planes in the four-segment mixing channel at an inlet flow velocity of 0.25 m/s, which corresponds to the *Re* of 25. The analyses of five mesh densities ranging from 3.577 × 10^5^ to 9.984 × 10^5^ are used and the two finest meshes give a negligible relative difference in their corresponding values that indicates they are mesh-independent. The values of maximum velocity inside the microchannel and the mixing index at the outlet section for the five mesh densities are also shown in Table 1. Finally, the mesh density with 8.663 × 10^5^ has been chosen for further investigation since the mixing indices at the specific location are almost the same and the numerical results are grid-independent. Also, the four-segment mixing channel is used unless noted otherwise.

## 3. Fabrication Process and Flow Visualization

For experimental characterization of mixing, the staggered microstructures with curved channels are fabricated by soft lithography techniques. For the mixing visualization, phenolphthalein and NaOH solutions are injected into the microchannel via two inlets, respectively. To verify the simulation results, a confocal microscope is utilized to monitor the mixing behaviors at the cross-sections of the mixing channel. Different values of *Re* are considered to investigate the effect of *Re* on mixing.

The flow device is created using a replica molding method. Initially, a silicon wafer is cleaned and dehydrated on a hotplate. An epoxy-based negative photoresist (SU-8) is spin coated on a silicon wafer. The photoresist is then pre-baked and soft baked on a hotplate. The channel pattern is fabricated by UV exposure. After development, the master is washed and baked to fix the photoresist. Once the mold is complete, the wafer is rinsed in deionized (DI) water and dried with nitrogen. The PDMS mixture which is thoroughly mixed with the prepolymer and curing agent in a 10:1 weight ratio is degassed with a mechanical vacuum pump to remove air bubbles. After pouring the PDMS mixture onto the SU-8 patterned master, the PDMS is then cured in a convection oven and the replicas are peeled off carefully from the master. The microchannels are made of PDMS molds and fabricated by conventional photolithography. The inlet and outlet holes are then punched out on the micromixer. Methanol is used as a surfactant to prevent the oxygen-plasma-treated PDMS replica and glass slide from being irreversibly bonded when aligned improperly. After surface oxidation and bonding, the designed microchannels which consist of twenty-two identical mixing segments are ready. The radius of curvature of the channel is 550 μm. The entrance and the outlet have a 0.01 mm^2^ square cross-section.

**Figure 3:**
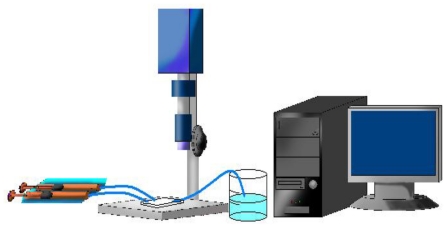
The setup of the measurement system.

Two different fluids are injected into the microchannels using a programmable syringe pump (KDS-101, kdScientific Inc., USA). Figure 3 shows the schematic of the experimental setup used to obtain mixing performances. The flow rates range from 0.003 mL/min to 0.3 mL/min corresponding to the *Re* from 0.5 to 50. The experimental setup for testing the performance of the fabricated micromixers is described as follows. Two syringes are loaded with 0.31 mol/L phenolphthalein and 0.33 mol/L NaOH dissolved in 99% ethanol. The NaOH solution possesses a pH value of about 13. Phenolphthalein solution, as a pH indicator, has a characteristic of changing color from transparent to purple at pH values greater than 8. As a result of the rapid reaction between phenolphthalein and NaOH, the interface between the two streams turns purple within a negligible time [17]. The working fluids first enter the inlet channels, flow through the micromixer, and finally exit through an outlet channel. Once a steady state is attained, an optical microscope (Eclipse 50i, Nikon, Japan) and CCD camera (DC80, Sony, Japan) are set up to observe the color changes inside the channel. Images are captured at each segment along the downstream direction. The captured digital images are converted into grayscale images that give the luminance of the image in 256 levels, and analyzed using software (MATLAB, The MathWorks, Inc., USA).

To verify the simulation results, a confocal microscope (Leica TCS SP2, Leica Corp., Germany) is utilized to monitor the mixing behaviors at the cross-sections of the mixing channel. One syringe is filled with fluorescent solution (99% DI water and 1% Rhodamine B, Fluka, Germany) while the other is filled with DI water only. The images of the fluorescent solution are excited at 543 nm with a He-Ne green laser and the signal of fluorescent emission can be detected in red (585 to 615 nm). Only the portion containing Rhodamine emits light when exposed to the laser. The fluorescence is monitored with a confocal microscope equipped with an air objective (10 ×/0.4, ∞/0.17/A). The XY cross-section is scanned with a resolution of 1024 × 1024, and the YZ cross-section is scanned with a resolution of 1024 × 240 (the total distance along the Z-axis is 100 μm with the confocal resolution of the channel height being 1 μm). The micromixers are designed for investigating the effects of various operational and geometric parameters on mixing.

## 4. Results and Discussion

### 4.1. Mixer Design

In this section, the design concept of the staggered Dean Vortex micromixer is introduced. A parallel lamination micromixer with two-dimensional curved rectangular channels is designed in our study. The centrifugal forces in curved rectangular channels cause fluids to produce secondary flows. A pair of counter-rotating vortices above and below the symmetry plane of the channel coincides with its plane of curvature. The split-and-recombination (SAR) structures of flow channels result in the reduction of the diffusion distance of two fluids. The impinging effects increase the mixing strength when one stream is injected into the other. The fluid properties in our simulations are diffusivity, *D*, of 4 × 10^−10^ m^2^/s, kinematic viscosity, *ν*, of 1 × 10^−6^ m^2^/s, density, *ρ*, of 1 × 10^3^ kg/m^3^ and used unless noted otherwise. The flow system is composed of several staggered three-quarter ring-shaped channels. The inner radius, *R_i_*, is 500 μm and outer radius, *R_o_*, is 600 μm. The inlet and outlet have square 100 μm cross-sections.

A particle trajectory is the path of a particle moving in a fluid. Being able to visualize this trajectory can be very helpful in understanding flow patterns and flow distribution. At a steady state, the streak lines coincide with the particle trajectories. These can be thought of as recordings of the paths where fluid elements flow over a certain period and are used to describe the nature of the flow field inside the micromixer. In this study, the particle trajectories are determined by integrating the vector equations of motion and obtain an explicit Lagrangian-type particle tracking scheme from CFD-ACE+^TM^ software. The integration is done by utilizing a fourth order Runge-Kutta scheme with adaptive step size, and the linear shape function [18] is used for the spatial interpolation of velocity.

**Figure 4:**
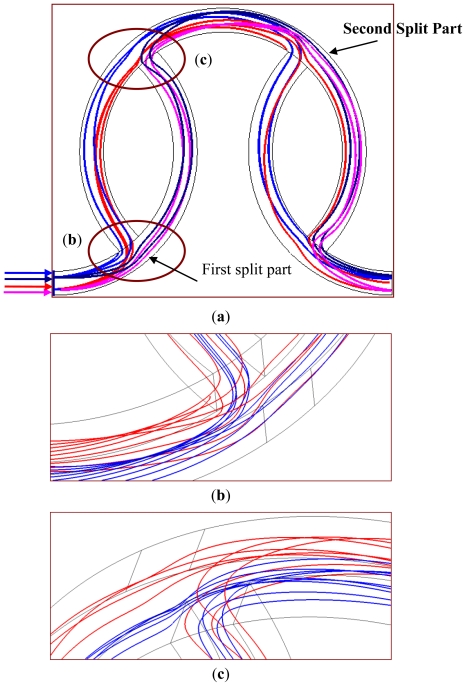
Particle trajectories of the staggered Dean Vortex micromixer from (**a**) a front view; (**b**) a vertical view near the split part and (**c**) a vertical view near the recombined part.

The particle trajectories inside the staggered curved channels at the first segment are shown in Figure 4. The inlet velocities of two liquids are set at 0.5 m/s, which correspond to a *Re* of 50. A front view of the particle trajectories through the mixing channels is depicted in Figure 4a. The red- and pink-colored trajectories represent species A, and the blue- and dark blue-colored trajectories represent species B. The red- and pink-colored trajectories stretch from the inlet, split into two streams, and merge back into one main stream. It is observed that species A and species B divide after the split part of channel, merge into one stream and re-allocate into two separate channels again. For species A, most of the red-colored trajectories move into the angled channel and only the red-colored trajectories near the outer region keep moving in the original curved channel after the first split part. However, after the second split part, some moves in the original curved channel and some shifts in the angled channel. This flow pattern also can be seen for the pink, blue and dark blue trajectories, respectively. Due to the SAR structures, the fluids switch between the original curved channels and the angled channels and wind along the downstream channels. When the SAR structures of the flow channels result in the reduction of the diffusion distance of two fluids, the mixing performance can be improved.

Figures 4b and c show the particle trajectories around the interconnection of two sub-channels (the region marked by an oval in Figure 4a) in the vertical views. In Figure 4b, both species split into two streams and then the contact interface between the two species can be increased. In Figure 4c, two streams merge and produce an impact around the recombined part. The main stream is deflected when the branch stream impinges on it. This impingement enlarges the mixing strength when one stream is injected into the other.

**Figure 5:**
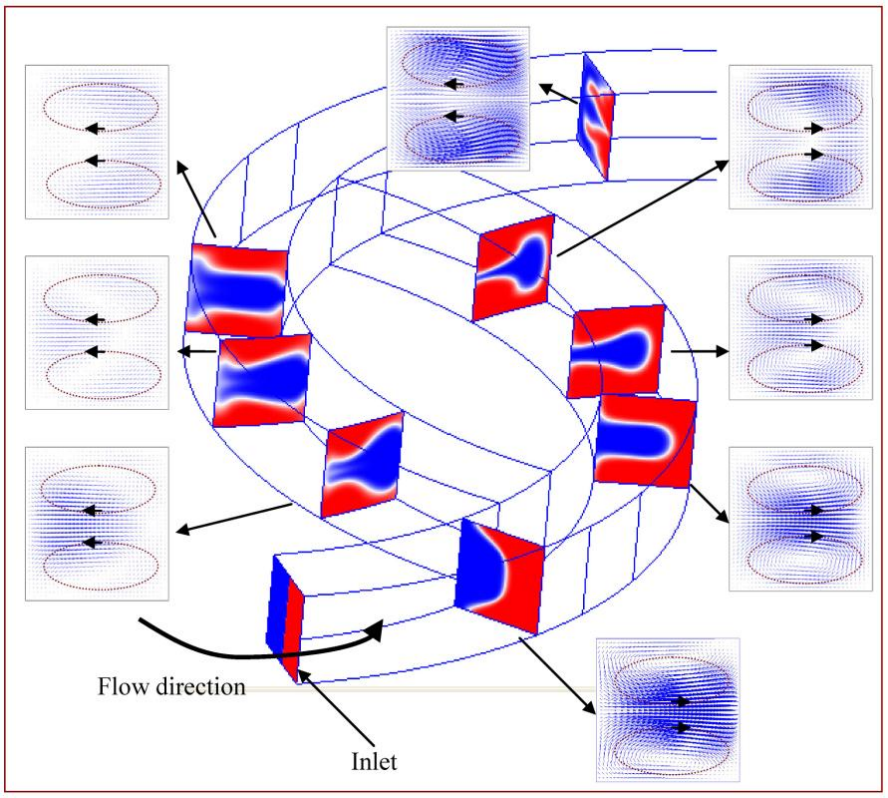
The vector planes and the mixing characteristics at eight cross-sectional areas along the downstream.

In an effort to understand two-fluid mixing inside the staggered Dean Vortex micromixer, the velocity vector planes and the cross-sectional concentration distributions are utilized to demonstrate the rotation of fluid flows. Several cross-sectional locations in the structure are expressed in Figure 5 at a *Re* of 50. The images are taken at the first half segment. The fluids flow into the inlet and progress counterclockwise along the curved channel. Dean flows are created in the channel of constant cross-section; one rotates counterclockwise above and the other clockwise below the symmetry plane of the channel (viewed from the direction of the arrow). One stream follows the original channel, and the vortices continue in the same direction. The other stream flows into the branch channel, and the vortices move clockwise above and counterclockwise below the symmetry plane of channel. In vector planes, the length of an arrow means the magnitude of the velocity vector. Compared with the vector planes between the two branch channels, a large amount of fluid tends to flow along the original curved channel (the right-hand side) and the rest of fluid moves into the angled channel (the left-hand side). The uneven split of the two fluids inside the staggered channels can be observed. Then the two channels are recombined, and the two streams merge into a single large one. After leaving this region, the vortices move clockwise above and counterclockwise below the symmetry plane of the channel. Similar flow characteristics can be seen along the downstream. However, the rotating directions of the created Dean vortices are switched. A significant amount of SAR vortex flows exist due to fluids flowing through the staggered curved channels. The ability to sequentially combine these flow regimes creates increased contact surface between the two fluids.

The cross-sectional concentration distributions are also used to demonstrate the mixing characteristics along the downstream channel in Figure 5. Fluid flows around a curved channel and the fluid near the centre experiences a larger centrifugal force than that near the top and the bottom. The fluid velocity along the central axis is the highest and is the most strongly affected by centrifugal forces. As a result, the liquid flowing along the central axis is thrust outward vigorously by inertial force, and the liquids near the top and the bottom flow inward. Thus, the vertical interface that crosses the central axis is distorted and bell shaped at this location. In the angled channel, blue-colored fluids are surrounded by red-colored fluids. It shows the lamellae of the two species and is accompanied by a corresponding increase in interfacial area. So the combination of SAR, Dean Vortex and a strong impact inside the channel significantly improves mixing.

**Figure 6:**
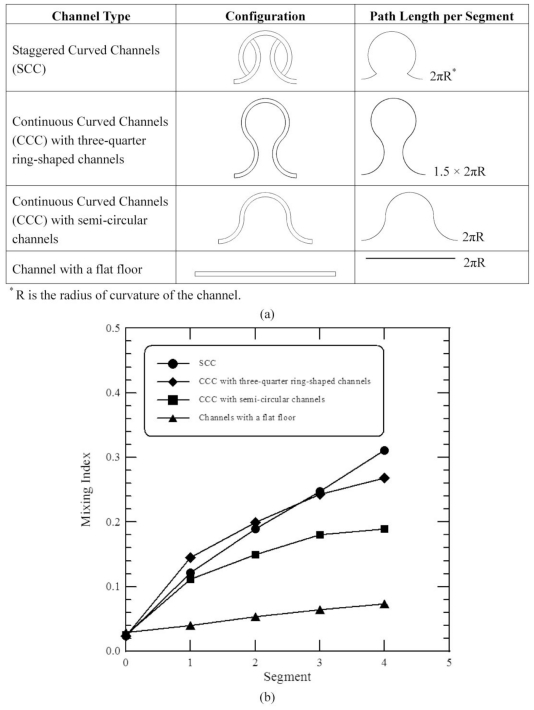
(**a**) The configurations of the four micromixers; (**b**) Comparison of the present numerical results with those of the previous design.

To evaluate the mixing performances of staggered curved microchannels, the results are compared with the previous work [12]. In every mixing segment of their design, the microstructure consisted of one three-quarter ring-shaped channel and two three-eighth ring-shaped channels (*i.e*., Continuous Curved Channels (CCC) with three-quarter ring-shaped channels). Each segment of our design is comprised of 3 staggered curved channels which are three-eighth ring-shaped and three-quarter ring-shaped (*i.e*., Staggered Curved Channels (SCC)). Our micromixer has the same separate microstructures as the previous design; however, the path length per segment is different, as shown in Figure 6a. In order to evaluate the mixing performances between the staggered curved microchannels and the continuous curved microchannels, a microstructure which is composed of one semi-circular channel and two three-quarter ring-shaped channels is also introduced (*i.e*., Continuous Curved Channels (CCC) with semi-circular channels). The path length per segment of the former structure is the same as that of our designed micromixer. This work also examines a microchannel with a straight section. The geometric design of the proposed CCC is modified and scaled to be a similar size to that of our mixer.

Figure 6b shows the mixing indices of a comparative analysis that was performed across four designs at an inlet flow velocity of 0.1 m/s and *Re* of 10, which corresponds to a Dean number (*K*) of 4.47 and a Péclet number (*Pe*) of 25000. The SAR channel and the fluid impact effect in the SCC improve fluidic mixing. The mixing performance shows improvement in Figure 6b. For the larger path length per segment of CCC with three-quarter ring-shaped channels, the mixing due to the secondary flows induced by the centrifugal force is larger than that of CCC with semi-circular channels. The effect of centrifugal forces on the mixing of CCC with semi-circular channels can be seen, and the mixing is better than that of the channel with a straight section. Lack of staggered geometries results in poorer mixing compared with the mixing index between SCC and CCC with the same path length per segment. It is found that little mixing occurs in the channel with a straight section. This difference is attributed to the creation of two vortices in the curved channels, which promote mixing. The general trend of present computational results agrees well with those of the previous results. Based on the same path length per segment it is clear that the mixing index of present geometry is considerably improved compared to the other designs.

In addition to mixing index, the pressure drop is also an important issue in the design of micromixers. The pressure drops in the microchannels with four segments are 5913.27 Pa, 2754.33 Pa, 3958.09 Pa and 3956.35 Pa for the CCC with three-quarter ring-shaped channels, the SCC, the CCC with semi-circular channels and the channel with a straight section, respectively. The SCC has the lowest pressure drop among these channels. Each segment of our micromixer is comprised of 3 curved channels with uniform width. The flow rate in each sub-channel is reduced compared to the main channel. Despite the existence of the additional bends and the presence of the Dean vortices, the pressure drop of the SCC is less compared to the channel with a straight section. Besides, the SCC has a higher mixing index than the straight microchannel from Figure 5b.

**Figure 7:**
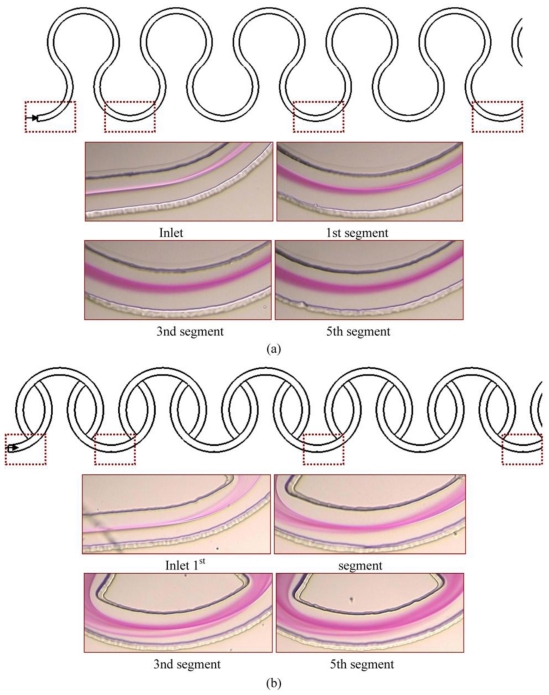
Mixing experimental results of (**a**) Continuous Curved Channels (CCC) with three-quarter ring-shaped channels and (**b**) Staggered Curved Channels (SCC), at the indicated positions, at the *Re* of 10. Only the reacted phenolphthalein at the interface between the phenolphthalein and NaOH streams shows as a purple color.

The front views of the images along the downstream direction were commonly used to qualitatively characterize the mixing performance of a micromixer. In our work, the front view of the color changes along the downstream direction are captured by a microscope and CCD camera with a graphic grabber system. When phenolphthalein meets and reacts with NaOH, it changes its color from colorless to purple so that the interface between two streams can be identified by a purple color. In the case of the continuous curved channels with three-quarter ring-shaped channels at *Re* equal to 10, as shown in Figure 7a, two streams flowing in parallel meet each other exactly at the central line of the channel and thus the interface is clearly observed near the inlet part of the channel. The thickness of the purple region increases along the downstream obviously due to the secondary flows. The color intensity indicates the level of mixing in the curved channel. With regard to Figure 7b, staggered curved channels, much more reacted solution stream passes through the inner half of the channel and the variation of the concentration distribution along the radical direction can be seen clearly. By the naked eye, we can obviously tell the superiority of the SCC over the CCC with three-quarter ring-shaped channels.

### 4.2. Parametric Study

**Figure 8:**
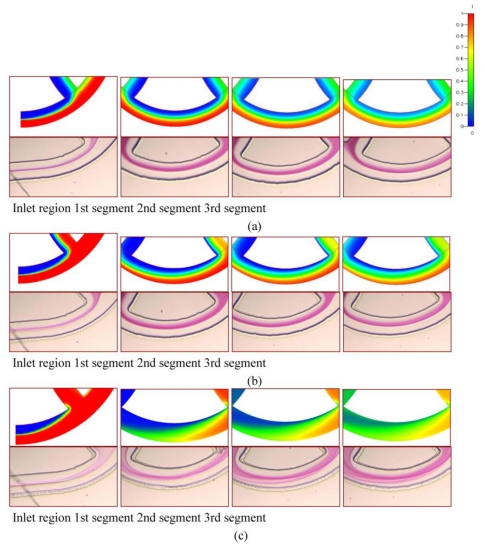
Numerical results (top) and reacted phenolphthalein images (bottom) for (**a**) *Re* = 1, (**b**) *Re* = 10 and (**c**) *Re* = 50 in different regions. The flow is from left to right.

The effects of various Reynolds numbers on the flow fields and the mixing characteristics inside the microfluidic system are examined. The comparisons of the mixing obtained from numerical analysis and experimental visualization are demonstrated. Figure 8 shows the numerical and the reacted phenolphthalein imaging results of staggered Dean Vortex micromixers in four representative regions along the downstream direction at various *Re*. The flow is from left to right. In Figure 8, the simulations shown on top are taken at the middle planes of channel height and the concentration distributions are demonstrated. The experimental images are shown on the bottom. The purple-colored streams represent the reacted phenolphthalein at the interface between the phenolphthalein and NaOH streams. In our work, two syringes are loaded with 0.31 mol/L phenolphthalein and 0.33 mol/L NaOH dissolved in 99% ethanol. The effect of the amount of phenolphthalein and NaOH on fluid properties is neglected. The fluid properties are set at the physical and thermodynamic properties of ethanol [19]. The fluid properties of ethanol used in Figure 8 are diffusivity, *D*, of 1 × 10^−9^ m^2^/s, kinematic viscosity, *ν*, of 1.52 × 10^−6^ m^2^/s, density, *ρ*, of 0.79 × 10^3^ kg/m^3^.

At *Re* = 1 in Figure 8a, the flows follow the contours of the curved channel. As the flow proceeds forward, a little more solution moves toward the inner part of the channel. The mixing of two fluids is only through molecular diffusion across the interface of the two liquids. However, Dean vortex flows can be created at *Re* greater than 10, as shown in Figure 8b. The interface surface deforms along the downstream. It shows that a more reacted solution stream passes through the inner half of the channel and the variation of the concentration distribution along the radial direction can be seen clearly. At a *Re* = 50 in Figure 8c, two fluids are folded into each other and split into multiple streams due to the large vortex flow combined with the SAR effect and the impact effect. Notably, because of the increases of the interfaces of the two fluids, reacted phenolphthalein streams are increased and mixing is improved. The results of the numerical simulations show that an enlarged interfacial surface area is increased considerably, especially for the case where *Re* is equal to 50. Due to numerical diffusion, simulations show better mixing than experiments. However, the comparison between the experimental data and numerical results shows a very reasonable agreement.

**Figure 9:**
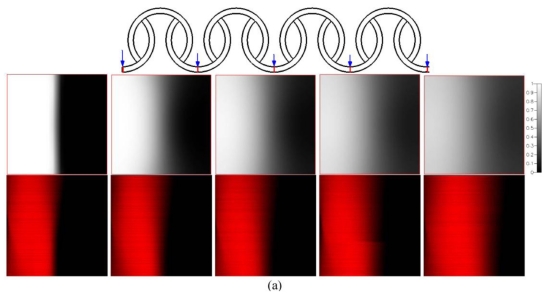

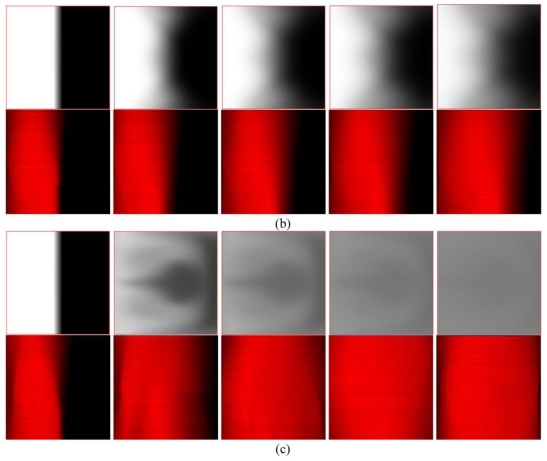
Numerical (top) and confocal images (bottom) for *Re* of (**a**) 1, (**b**) 10 and (**c**) 50 at five cross sections.

Figure 9 shows cross-sectional mixing behaviors in the staggered Dean Vortex micromixers at five locations observed by means of a confocal microscope. The mixing results are taken at five representative positions, such as the inlet, the first, the second, the third and the fourth segments beyond the inlet in the downstream direction, marked by red lines. Around the inlet region of the SCC, only the half-zone containing Rhodamine shows a bright image as a result of laser scanning. The interface between streams is distorted as it moves downstream. The fluid near the centre experiences a larger centrifugal force than that near the top and the bottom. Consequently the liquid flowing along the central axis is forced outward (from left to right), and the liquids near the top and the bottom flow inward (from right to left). Thus the stretching of the interface can be presented and enhance mixing. We found that fluids are mixed well and the interface is distorted at a high *Re*. As demonstrated by the confocal microscope images in Figure 9, the staggered Dean Vortex micromixer can be regarded as a well-designed passive micromixer to achieve almost complete mixing in a manageable length of channel. The results are also compared with the numerical results and a similar trend can be shown.

**Figure 10:**
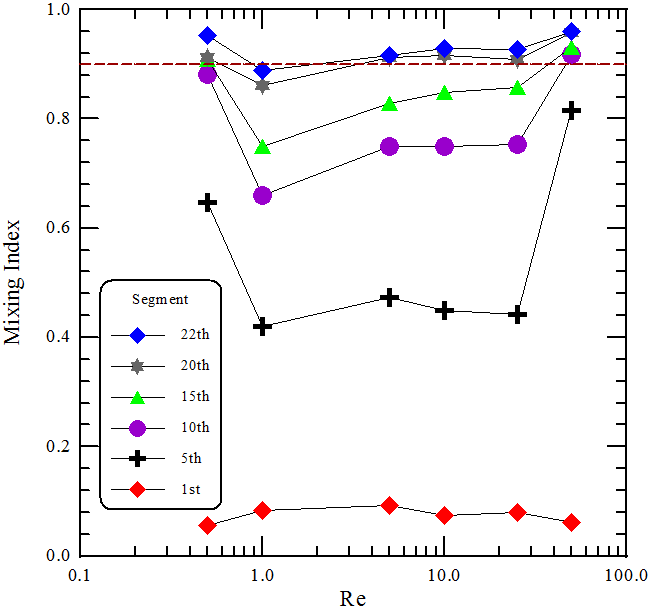
Plot of the mixing index at each observation segment. The dashed line shows the mixing index of 0.9.

The mixing performance of two fluids, phenolphthale and NaOH dissolved in ethanol, at specific segments is demonstrated in Figure 10. The influences of various *Re* on the mixing index is depicted. Each normalized intensity is marked by the different symbols is plotted at the abscissa corresponding to different *Re*. At low *Re*, 0.5 < *Re* < 1, viscous forces in the fluid are larger than inertial forces; thus, inertia can be neglected. Mixing within the stated range of *Re* is dominated by pure molecular diffusion. The time of contact between the two fluids is sufficiently long enough to generate significant mixing when *Re* is small. The mixing index then decreases as the *Re* increases within the range of 0.5–1. As the *Re* rises to 5, the inertial force increases but the centrifugal force acting on the fluids is still weak. The secondary flow is not strong enough to induce sufficient rotation. The mixing index increases with increasing *Re* when *Re* is greater than 10; the increased inertial force enlarges the contact area between the two fluids. From the preliminary studies on the micromixer with curved channels, Dean vortex flows can be created at *Re* greater than 10. Thus the deformation of interfacial lines is profoundly affected by secondary flow and related to the mixing characteristics. When the *Re* is equal to 50, the fluids experience several segments of rotation, SAR and impact consequence. Accompanied by a corresponding increase in interfacial area, good mixing at the channel end has been achieved.

The dashed line in Figure 10 represents a mixing index equal to 0.9 which denotes that mixing fluids are in a well mixed status. The mixing length is the channel length required for achieving the mixing index of 0.9. And it is a distance that a fluid will keep its original characteristics before dispersing into the surrounding fluid. By means of the mixing length, the required channel length of the micromixer can be demonstrated. The resulting mixing lengths are at the tenth segment, over the twenty-second segment, twentieth segment, seventeenth segment, sixteenth segment, and sixth segment at *Re* of 0.5, 1, 5, 10, 25 and 50, respectively. The mixing index at *Re* of 50 reaching to 0.9 at the sixth segment is not shown in Figure 10. These correspond to the downstream distances of 35 mm, 77 mm, 70 mm, 60 mm, 56 mm, and 21 mm. At the *Re* of 50, the achieved mixing length (*i.e*., 21 mm) is 4 times shorter compared to the case where the *Re* equals 1 (*i.e*., 77 mm).

### 4.3. Micromixer Optimization

The performance parameter defined for this study is the mixing quality. The selection of the appropriate design variables that affect mixing quality is one of the most important aspects of design optimization. Among the geometric variables shown in Figure 1a, the angle between the lines from the center to the two intersections of two consecutive curved channels, *θ*, and the angle between two lines of the centers of three consecutive curved channels, *ϕ*, are selected as the design variables for optimization. The effect of various inner radius and outer radius on mixing is included and related with the design variable, *θ*. It means that the effect of various channel widths (the difference between the inner radius and the outer radius of channels) on mixing is also associated with the design variable, *θ*. The design space is selected through the method of steepest ascent [20].

The first step in obtaining the optimum response settings is to explore the region around the current operating conditions to decide what direction needs to be taken to move towards the optimum region. A first order regression model is utilized at the current operating conditions because the operating conditions are normally far from the optimum response settings. The range of the factors for this design are chosen to be (80°, 90°) for *θ* and (10°, 30°) for *ϕ*. The unreplicated 2^2^ design is augmented with one run at the center point (85°, 20°) to check for model adequacy. Then the first order regression model at an inlet flow velocity of 0.25 m/s, which corresponds to the Reynolds number of 25, is expressed as:

$$MI=0.418+0.015\times \left(\frac{\theta -85{}^{\circ}}{10{}^{\circ}}\right)+0.036\times \left(\frac{\theta -20{}^{\circ}}{20{}^{\circ}}\right)$$

where the *p* values of two design variables are less than 0.05. *p_θ_* and *p_ϕ_* are 0.004 and 0.001, respectively. And the *p* value of the interaction of two design variable, equal to 0.8215, is larger than 0.05. So the factors affect the response significantly but their interaction does not affect the response.

**Figure 11:**
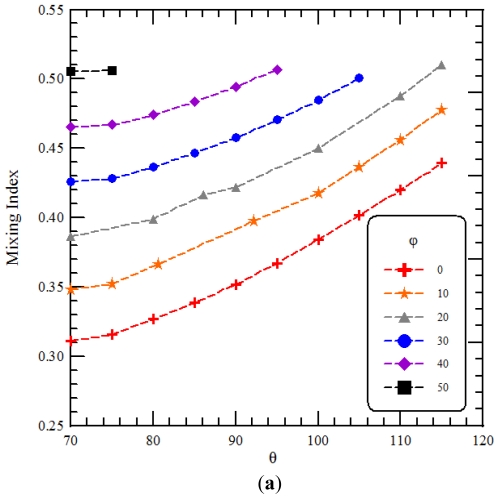

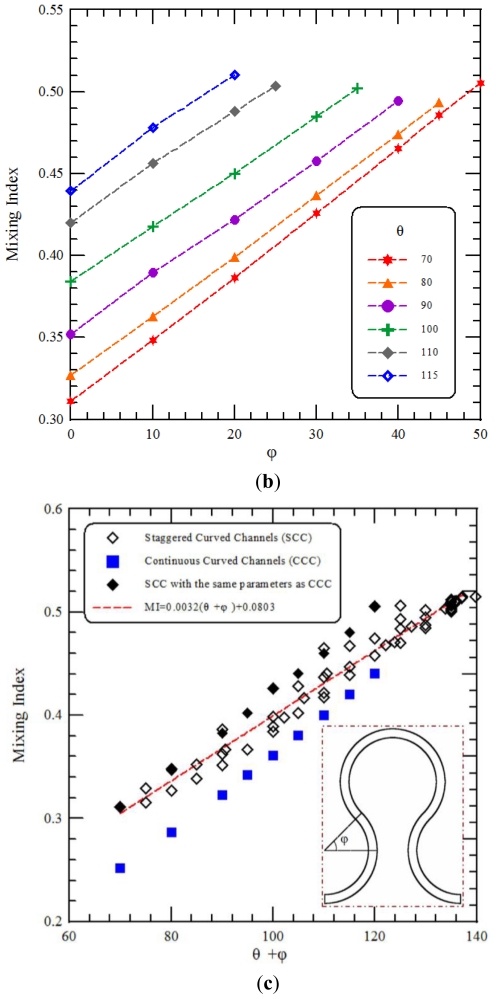
(**a**) The relationship between *θ* and the mixing index, (**b**) the relationship between *ϕ* and the mixing index and (**c**) a linear relationship between the sum of *θ* and *ϕ* and the mixing index.

Extensive numerical simulations were conducted by changing the physical characteristics of *θ* and *ϕ*. Figure 11a,b show the relationship between the design variable and the performance parameter and depict the mixing index as a function of *θ* and *ϕ*, respectively. *Re* of 25 are considered. Results show that the mixing index exhibits a monotonic increase with the increasing of *θ* and *ϕ*. Compared to the first order regression model, we can also find that a similar trend is shown and the mixing index increases as *θ* or *ϕ* increases. Figure 11c shows a linear relationship between the mixing index and the sum of *θ* and *ϕ* of staggered curved channels. The linear relationship is given by

MI=a(θ+ϕ)+b

where *a* is around 0.003166, and *b* is around 0.08226. It can be found that the maximal mixing index is related with the maximal value of the sum of *θ* and *ϕ*. The effect of *ϕ* on the mixing index of continuous curved channels with the same path length per segment as the SCC is also shown in Figure 11c. The intersection of two consecutive curves of continuous curved channels is located at a point, and *θ* does not exist. We take *θ* equal to 70° as a base reference. Results show that the mixing index of continuous curved channels (marked by blue square points in Figure 11c) increases with the increasing of *ϕ* and is less than that of staggered curved channels with the same values of the sum of *θ* and *ϕ* (marked by black diamond points in Figure 11c).

**Figure 12:**
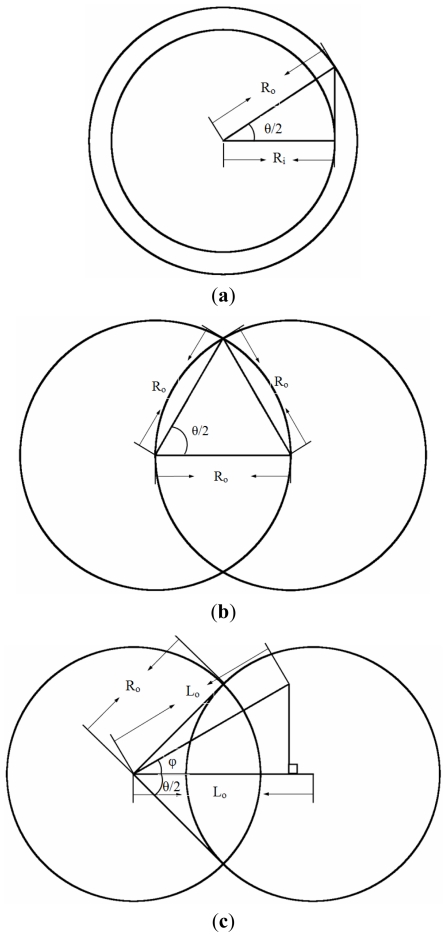
The geometric constraints of the staggered curved channels. (**a**) The minimum value of *θ*, (**b**) the maximum value of *θ* and (**c**) the maximum length between the centers of the consecutive circles.

The maximal mixing index is associated with the maximal value of the sum of *θ* and *ϕ* from Figure 11c. And these design variables, *θ* and *ϕ*, are determined by the inner and outer radii of the curved channel and the distances between the centers of three consecutive curved channels. In order to perform the optimization of staggered Dean Vortex micromixers, the relationship between the ratio of the inner radius to the outer radius and the design variables should be found. Due to the geometric constraints of staggered curved channels, the ranges of *θ* and *ϕ* are limited. In Figure 12a, the minimum value of *θ* can be determined by

$$\frac{\theta }{2}\geq {\text{cos}}^{-1}\left(\frac{R_{i}}{R_{o}}\right)$$

where the minimum value of *θ* is dependent on the ratio of the inner radius to the outer radius of the curved channel. It also means that the design variable, *θ*, is chosen and the effects of various inner radii and outer radii on mixing are included. For example, when *R_i_* is 500 μm and *R_o_* is 600 μm, then the minimum of *θ* is about 67.1°. In Figure 12b, the maximum value of *θ* can be determined by

$$\frac{\theta }{2}\leq 60{}^{\circ}$$

where the maximum value of *θ* is 120° and is a constant.

The maximum length between the centers of consecutive circles, *L_o_*, is shown in Figure 12c. The relationship between *L_o_* and *R_o_* is expressed as

$$L_{o}\;\text{cos}\;\phi \geq R_{o}\;\text{or }2\times R_{o}\times \text{cos}(\frac{\theta }{2})\text{cos}\;\phi \geq R_{o},\;\text{and then cos}\left(\frac{\theta }{2}\right)\text{cos}\;\phi \geq \frac{1}{2}$$

where the maximum value of *θ* or *ϕ* can be determined by the above inequality. From Figure 11c, the maximal mixing index is related with the maximal value of the sum of *θ* and *ϕ*. The maximal value of the sum of *θ* and *ϕ* can be found by following. An auxiliary function *f* (*θ, ϕ*) = *θ* + *ϕ* is set and $\phi ={\text{cos}}^{-1}\left(\frac{1}{2\;\text{cos}(\theta /2)}\right)$ is introduced into the auxiliary function. Then the maximal value of the sum of *θ* and *ϕ* can be solved numerically and is around 139.82° where *θ* is around 106.21°, *i.e.*, *R_i_*/*R_o_* = 0.601, and *ϕ* is around 33.62°. Therefore, the maximal mixing index can be found for any staggered Dean Vortex micromixers when

$$\{\begin{array}{ll} 1>R_{i}/R_{o}>0.601, & \theta \approx 106.21{}^{\circ}\;\text{and }\phi \approx 33.62{}^{\circ}\\ 0.601>R_{i}/R_{o}>0.5, & \theta =2\;{\text{cos}}^{-1}\left(\frac{R_{i}}{R_{o}}\right)\;\text{and }\phi ={\text{cos}}^{-1}\left(\frac{1}{2\;\text{cos}(\theta /2)}\right)\\ 0.5>R_{i}/R_{o}, & \theta \;\text{and }\phi \;\text{do not exsit} \end{array}$$

This relationship can help researchers who are interested in designing staggered Dean Voxter micromixers.

**Figure 13:**
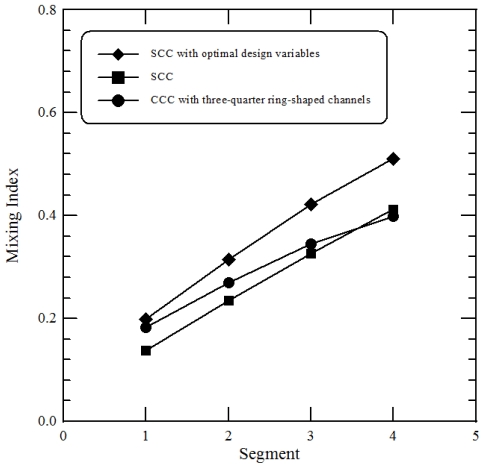
Comparison of the present numerical results with those of the previous design. These are Staggered Curved Channels with *θ* equal to 106.2° and *ϕ* equal to 33.6°, Staggered Curved Channels with *θ* equal to 90° and *ϕ* equal to 0° and Continuous Curved Channels with three-quarter ring-shaped channels.

Figure 13 shows the results of a comparative analysis at a *Re* equal to 25 that are performed across the three designs, viz., staggered curved channels with *θ* equal to 106.2° and *ϕ* equal to 33.6°, staggered curved channels with *θ* equal to 90° and *ϕ* equal to 0° and continuous curved channels with three-quarter ring-shaped channels. It is clear that the mixing is considerably improved throughout the channel length by optimization. The values of the mixing index achieved at the exit for the optimal and the other geometries represent a relative increase of about 12.5% in mixing.

## 5. Conclusions

In this paper, we have presented a study of fluid mixing in staggered Dean Vortex micromixers. The existence of the secondary flows, the split-and-recombination (SAR) structures of flow channels and the impinging effects result in increased mixing strength. On the basis of this principle, a parallel lamination micromixer composed of several staggered three-quarter ring-shaped channels has been optimized and fabricated in our study. Three-dimensional computational fluid dynamics simulations are performed to investigate the mass transfer and the fluidic behavior in a curved microchannel. The vector planes, the cross-sectional concentration distributions and the particle trajectories are utilized to examine the mixing and the flow characteristics inside the staggered curved microchannel. The uneven split of two fluids inside the staggered channels improves mixing performance. The effects of various Reynolds numbers and channel configurations on mixing performances are investigated in terms of the experimental mixing results through a pH indicator using an optical microscope and fluorescent particles via a confocal microscope. The results indicate that for low Reynolds numbers (<5) diffusion is the primary mechanism by which mixing occurs. At Reynolds numbers greater than 10, secondary flows come into play and the lamellar formation contributes to increased levels of mixing. At a *Re* of 50, the mixing length of 21 mm can be achieved in a distance 4 times shorter than when the *Re* equals 1 (*i.e*., 77 mm). The simulation results are in reasonable agreement with the experimental measurements. The geometric optimization of a staggered Dean Vortex micromixer has been performed. The mixing index has been taken as the performance parameter with two design variables, viz., the angle between the lines from the centre to two intersections of two consecutive curved channels and the angle between two lines of the centers of three consecutive curved channels. Therefore, the maximal mixing index can be found for any staggered Dean Vortex micromixers. The optimized micromixer with staggered curved channels shows an improvement in the mixing performance of about 12.5% relative to the previous design with continuous curved channels.

The major goals of this paper are to investigate the physical insights of the flow characteristics and the mixing performances in staggered Dean Vortex micromixers. The readers are referred to recommended literature [10,11] for a detailed discussion about the effects of the curvature designs on mixing. Our future work is to optimize the staggered Dean Vortex design by systematically integrating a CFD package with an optimization methodology based on the use of experiment design. Two different components of the performance parameters, mixing index and pressure drop, will be employed to optimize the micromixer.

## Figures and Tables

**Table 1:** The analysis of the grid size independence.

Number of Nodes	Maximum Velocity	Mixing Index	Relative Difference in Maximum Velocity (%)	Relative Difference in Mixing Index (%)
3.577 × 10^5^	1.01057	0.794	-	-
4.992 × 10^5^	1.01643	0.741	0.577	6.675
6.582 × 10^5^	1.02036	0.715	0.385	3.509
8.663 × 10^5^	1.02224	0.701	0.184	1.958
9.984 × 10^5^	1.02270	0.695	0.045	0.863
